# Sanitary Science

**Published:** 1875-01

**Authors:** 


### SANITARY SCIENCE.

A distinguished Scotch physician recently declared in Glasgow that the causes of mortality were constantly increasing and that the mortality itself would as surely increase unless hygienic improvements kept pace with the causes of death. The question to be asked is, what are proper sanitary arrangements? There is no mystery in the matter. The problem is simple enough; it is only in the application that complications arise. A great part of sanitary science can be summed up in one word, cleanliness. Clean houses, clean water, and clean air, those are the enemies which no epidemic can resist. This theory may not be "as old as the hills," but is surely as old as human knowledge. There are no better rules for disinfecting foul smells than those of Ulysses as described in Homer. Hercules, too, was one of the oldest and most thorough of sanitarians, and Moses was among the most practical. The hygienic laws supplementary to his ten moral commandments were full of wisdom. Religious purifications are in the main hygienic precautions, and it is clear that the ancients knew as well as the moderns the main conditions of public health. The substance of all our sanitary science accumulated by ages, may be summed up in the pregnant advice of the prophet, "Wash and be clean." Not that the most profound inquiries into the origin of disease are to be undervalued. When we found that the virus of small-pox reproduced small-pox only, and that of scarlet fever bred scarlet fever only, we were as much inclined to refer their origin to a specific organism as to attribute a puppy to a dog or thistledown to a thistle. The popular tradition that the body changes itself only once in seven years is nonsense. The fact is, all the particles of the body change every six weeks. But the researches on the origin of disease, though vastly important, are scarcely yet within the domain of practical application. These bodies of low organized types are always associated with foulness. But whether putrid emanations are the result of the growth of these organisms, or whether the emanations form the only soil in which they can grow, no one can tell with certainty. Nor does it perhaps matter, practically, the certain thing being that if filth were prevented, none of these entozoa would remain permanently. What we mean by cleanliness is not merely personal ablution, but an uncompromising war with uncleanliness of all kinds. But to neglect of personal cleanliness epidemics might well be due. For a thousand years after the civilization of the Egyptians, the Jews, the Greeks, and the Romans faded, there was not a man or woman in Europe that ever took a bath. Hence arose the wondrous epidemics of the middle ages, which cut off one-fourth of the population of Europe—the spotted plague, the black death, the sweating sickness, and the terrible mental epidemics which followed in their train—the dancing mania, the mewing mania, and the biting mania. The monks made no little mischief, imitating the foul habits of the hermits and saints of early Christian times, and the association of filth with religion led men to cease to connect disease with uncleanliness, and to resort to shrines and winking virgins for cures of maladies produced by their own physical and moral impurities.

Even now we do not understand thoroughly the processes of purification, whether natural or artificial, but we know how the atmosphere contains in itself the power of freeing itself from corrupt matter: by motion and by the attacks of oxygen upon all such matter, burning up the products of decay, and turning organic into inorganic matter. But we only half learn the lessons nature teaches us. We do not allow garbage to be thoroughly oxidized, but dig holes and store it up where the air cannot penetrate, and where the greatest facilities are offered for injurious putrefaction. Or we poison rivers with it, pouring in far more matter than can be reached by natural agents. The only safe rule is to allow none at all to be poured in. Our rivers should never be used for drainage either by manufacturers or by cities and towns. We must ventilate and we must stop over-crowding in dwellings, this last being—next to alcohol—the most prolific source of disease and crime. The learned Dr. Playfair, of Scotland, computes that there are unquestionably not less than four million two hundred thousand cases of preventable illness in Great Britain. In the United States there are unquestionably half as many. We can greatly lesson the number, as well as the deaths resulting therefrom, by educating the people up to better, cleaner, more temperate, and nobler habits of thought and life. To this end we labor.

---

Brain Food.—Scientific men seem to have arrived at the fact that the brain is largely fed on food containing phosphorus, and that the great purifier of the blood, the food on which the lungs feed, is the oxygen of the air. But if we feed the lungs on pure oxygen, or eat unadulterated phosphorus as a means of nourishing the brain, we should die in a day. Nature must do our mixing. The food we eat, if properly digested, will give us all the phosphorus needed and the out-door air all the oxygen we require.

